# Designing a tool to support patient and public involvement in research projects: the Involvement Matrix

**DOI:** 10.1186/s40900-020-00188-4

**Published:** 2020-06-16

**Authors:** Dirk-Wouter Smits, Karen van Meeteren, Martijn Klem, Mattijs Alsem, Marjolijn Ketelaar

**Affiliations:** 1 Center of Excellence for Rehabilitation Medicine, UMC Utrecht Brain Center, University Medical Center Utrecht, and De Hoogstraat Rehabilitation, Utrecht, the Netherlands; 2 ‘Denker in Beweging’, Organization for Action Research Involving Parents and Children, Terheijden, the Netherlands; 3 ‘OuderInzicht’, Parent Organization for Increasement of Parent Involvement in Research, Amsterdam, the Netherlands; 4 BOSK, Association of People with congenital disabilities, Utrecht, the Netherlands; 5 Department of Rehabilitation Medicine, Amsterdam University Medical Centers, location AMC, Amsterdam, the Netherlands

**Keywords:** Patient involvement, Research, Projects, Roles, Expectations

## Abstract

**Background:**

Interest in patient involvement in research is growing. Research should rather be ‘with’ or ‘by’ patients, and not only be ‘about’ or ‘for’ patients. Patients’ active involvement in research is not self-evident and special efforts have to be made. If we make efforts towards patient involvement, it could contribute to even more relevant projects with an even greater impact. In this paper we describe the process of development of a tool to support patient involvement in research projects.

**Methods:**

The tool development was done in a co-creation of experience experts (patients and their parents/relatives) together with researchers. We used a participatory method in an iterative process comprising three consecutive stages. First, the purpose for the tool was explored, using focus groups. Second, the main ingredients and conceptualization for the tool were determined, using a narrative review. Third, the so-called Involvement Matrix was formalized and finalized using various expert panels.

**Results:**

A conversation tool was developed, through which researchers and patients could discuss and explain their roles of involvement in a research project. This tool was formalized and visualized as a ‘matrix’. The so-called Involvement Matrix describes five roles (i.e., Listener, Co-thinker, Advisor, Partner, and Decision-maker) and three phases (i.e., Preparation, Execution, and Implementation) and includes a user’s guide.

**Conclusion:**

The Involvement Matrix can be used prospectively to discuss about possible roles of patients in different phases of projects, and retrospectively to discuss whether roles were carried out satisfactorily. Sharing experiences with the Involvement Matrix and evaluating its impact are the next steps in supporting patient involvement in research.

## Plain English summary

There is increasing interest in patient and public involvement in research. However, tools are lacking to help patients and researchers understand and guide their involvement in research projects. In an iterative, collaborative process with researchers, clinicians, patients and relatives, we developed the ‘Involvement Matrix’. The Involvement Matrix is a tool that distinguishes different roles of involvement at different phases of research projects. The roles that are described in the matrix are Listener, Co-thinker, Advisor, Partner, and Decision-maker. Researchers and patients can discuss the desired roles in different phases of research projects beforehand, and evaluate these roles during the project and afterwards.

## Background

There is growing consensus about the importance of patient and public involvement (PPI) in research, and initiatives and publications on this topic are increasing rapidly [1, 2]. PPI in research is being defined as “research being carried out ‘with’ or ‘by’ members of the public rather than ‘to’, ‘about’ or ‘for’ them” [3]. ‘Public’ in this definition includes patients, potential patients, carers and people who use health and social care services, as well as persons from organisations that represent people who use services.

Arguments for PPI come down to three key premises [4, 5]. First, from a philosophical perspective, patients and public should be involved in deciding the research that concerns them and those in similar life circumstances. These arguments often refer to the slogan “Nothing about us without us” [6]. People have the right to participate in decisions that will (eventually) affect their lives [7]. Second, from a political perspective, taxes are used to subsidise the research, and therefore patients and public have a democratic right to influence what is supported. Funding agencies increasingly value the power of commitment of end-users in the implementation of results of research. Third, from a quality perspective, active involvement concerns the contribution of experiential knowledge. Patients and public possess unique knowledge and specific experiences. They have ideas about relevant research questions, about research designs and research procedures that are acceptable to them, and they provide a complementary perspective on findings and their interpretation.

Although publications and journals on the topic of PPI are increasing rapidly, a robust evidence base for the impact of PPI in research is still lacking. Many publications concern the process of PPI in general and only few publications take up the actual impact of PPI in research. Currently, most findings about effects and challenges are descriptive and based on anecdotal experiences; though some general patterns can be observed. For the research itself, reported benefits include prioritisation of research questions with relevance and importance to patients and public; identification of issues and details that researchers may not have been initially aware of (e.g. focus on outcomes that matter for the individuals concerned); study protocols and interventions being more acceptable and sustainable; recruitment and advertising materials being more age-appropriate and accessible; and dissemination having an extended reach [8, 9]. So far, there is some evidence of the impact of PPI improving enrolment and retention in clinical trials [10]. People are more likely to take part in studies that address their own priorities, and that fit their lives and family situations. In addition, not only for the research itself but also for patients and public, several benefits have been reported. They include patient empowerment, with increased confidence, self-esteem, enhanced knowledge, skills, and access to decision-making; increased awareness of health issues and sense of control over health service involvement; greater responsibility and independence; and increased likelihood of being involved in community programs after completion [8, 9].

However, the positive effects of PPI in research are not always easy to attain [8, 9, 11–14]. An important challenge that has been reported includes power imbalance, potential conflicts, and disagreement about roles and foci of interest [8, 9]. It can be difficult for researchers to truly share power when universities are often the main recipients of research grants and academics are ultimately accountable for how the money is spent [15]. From the perspective of patients and public, differences in educational levels and research expertise could result in disappointment, frustration and powerlessness. Another challenge in PPI is the issue of tokenism, with only symbolic efforts to involve end-users, without true openness to the ideas of others [8, 16]. Finally, a common finding is the inconsistency of involvement in the various stages of the research process. End-users are most often involved only in the early stage and/or in the final stage of research [11].

The current state-of-the-art on *how* to involve patients in research is largely experience-based [12, 14]. Recently, an overview of principles and best practice activities to support PPI efforts has been published, including an overarching foundational framework for partnerships between patient stakeholders and researchers [2]. Basic principles that have been found to be crucial in PPI concerned Respect & Equity, Trust, and Empowerment, including role clarity and clarity on expectations as a central prerequisite. Different expectations about roles and responsibilities can disrupt even the most promising PPI initiative [17]. Also from our own experiences in research, in which we involved patients, relatives, young persons with disabilities, and parents [18, 19], we felt PPI could particularly benefit from tools facilitating discussion and clarity about roles and expectations. In general, the value of conversational approaches between researchers and patients is underscored in more and more publications [20–22].

To our knowledge no specific tools are currently available to support the conversation and discussion about roles and expectations on the individual level, aiming for authentic and sustainable partnerships in research. Moreover, current tools and activities intending to facilitate aspects of PPI often have been developed by researchers themselves, without involving patients and public, while implementation of tools and activities will benefit highly from involvement of end-users in the development of it. Therefore, we aimed to design a tool that supports PPI in research projects, focusing on clarifying roles and expectations. This paper describes the process of developing the tool up to prototype, and its first application.

## Methods

For the tool development, a participatory method was used, through which meaningful dialogues were enabled between patients and researchers [23, 24]. Since the intended tool should not only be useable for researchers but also for patients, we developed the tool in an iterative co-creation process [25].

### Collaborative group

The ‘we’ in this paper comprises a collaborative group, including experience experts (i.e., patients and their parents/relatives), representatives of the patient organization BOSK (Dutch association of persons with a physical disability), health care professionals (such as doctors), and researchers.

### Sampling of participants

Within this participatory project, the type of sampling of participants was best described as ‘iterative sampling’. In this approach, what emerges from analyses in one stage shapes decisions for sampling and also for analyses in a following stage [26]. Thus, an open process of co-creation was facilitated. The process was not fully predetermined, but was open for contribution from all participants.

### Stages of tool-development

Three stages were planned in advance. Although the exact processes and outcomes within each of the three stages were deliberately left uncertain, we did have loosely formulated goals, with methods emerging naturally during the process. *Stage 1* was about finding a common ground (using focus groups): what kind of tool do we need as collaborators and for what purpose? *Stage 2* concerned determining the main ingredients and conceptualization of the tool (using a narrative review). *Stage 3* included formalizing the tool (using focus groups again), so that it could be used easily in practice.

### Setting and procedures

From January 2017 to December 2018, the project was conducted in the Netherlands by three main institutions: the Center of Excellence for Rehabilitation Medicine Utrecht (research center), the BOSK (patient association), and Stichting OuderInzicht (foundation to support parent involvement in research). Ethical procedures were not applicable. This participatory project aimed to design a (non-medical) tool without imposing actions on participants and therefore did not fall under the scope of the Dutch Medical Research Involving Human Subjects Act (WMO). Still, as some of the involved participants were patients or representatives, we complied with fundamental principles of good ethics in research with patients, such as respecting autonomy and keeping them informed about decisions [27].

## Results

### Stage 1. Exploring the common purpose: a conversation tool

The first step was to explore our common ground and purpose. Therefore, we organised focus groups with physical presence of five parties: patients (*n* = 3), relatives/parents of patients (*n* = 2), representatives of the patient organisation (*n* = 3), doctors (*n* = 1), and researchers (*n* = 3). In these focus groups, we found out that there was a mutual need for a tool that promotes real collaboration with patients in future research projects. In particular, the focus groups showed that the intended tool should help discussing two important topics: 1) the *roles* that an experience expert could play on the individual level in a research project and 2) the *expectations* that both the experience expert and the researcher have of concrete collaboration in a research project.

With the emergence of these two topics (i.e., Roles and Expectations), the focus groups put forward that the intended tool should not merely have an analytical purpose. In other words, the tool should not just measure (or analyse) PPI but rather create (or produce) it. The kind of tool that we all believed could be very helpful and could make a real impact was a ‘conversation tool’: preferably a visual aid – for instance a picture – that could be talked about together and that could thus guide the dialogue between the researcher and patient. Directed by shared experiences from everyone’s practice, the idea of a conversation tool became an important common ground for further tool development.

### Stage 2. Determining the tool’s ingredients: distinct roles of involvement

Now that the joint preference for a conversation tool had been expressed, the specific ingredients and conceptualization of the tool could be determined. The basic idea was that Arnsteins ‘ladder of participation’ [28] might be useful as the foundation of a tool to support PPI in research. This ladder of participation, namely, is often referred to when searching for information on PPI [29–31].

Inspired by the initial focus groups, the researchers (*n* = 3) in our collaborative group performed a narrative review [32] for answering the question which literature-based principles could be part of our tool. To start, the narrative review was performed with the key-word search ‘Arnstein‘, ‘participation’ and ‘ladder’, using Google and also the PubMed database. Thereafter, to put the review a bit more in perspective of research projects, the key words ‘involvement’, ‘engagement’, ‘research’ and ‘roles’ were added. This basic review concerned published (e.g. scientific articles and books) as well as grey literature (e.g. policy papers and project reports).

From the collected information, we learned that many authors after Arnstein have further developed the participation ladder [33], and also for the purpose of patient involvement in research projects (e.g., [29–31]). This development for research purposes was particularly seen from about 2010 and later. Although the given levels of participation – or involvement – vary somewhat in quantity (i.e., number of levels) and quality (i.e., description of levels), the divisions used by different authors are broadly in line. Secondly, we learned that more and more literature sources seem to avoid the term ‘levels’ and instead are using ‘roles’ [13, 34]. In addition, many sources prefer putting the ladder horizontally instead of vertically. Therewith they emphasize equality of the roles, while avoiding a hierarchical approach [29, 35]. A third lesson learned from the narrative review was that, regarding patient involvement in research projects, only those roles seem to be appropriate that actually stand for working together on a project [36]. Here, the role of study respondent should be left out of consideration and the same for decorative roles (i.e., being present without a real understanding of the project).

Overall, it became clear that Arnstein’s ladder of participation contained useful principles but needed several adjustments in view of designing a practical tool intended for research projects. Table 1 presents an overview of the main principles, suggested adjustments and related ideas derived from our narrative review.

**Table 1:** Principles, adjustments and ideas (derived from Arnsteins ladder of participation) that are useful for designing a tool supporting PPI in research projects

Principles	Suggested adjustments	Related ideas and solutions
Ladder for involvement	About *structure*: less focus on hierarchy	Horizontal structure
Separate levels	About *terminology*: roles instead of levels	Specific roles for patients’ involvement in research and not merely researchers’ involvement
Quantitative division	About *reduction*: only roles that imply working together (no decorative roles)	Five roles of involvement: listener, co-thinker, advisor, partner, and decision-maker
Qualitative division	About *description*: clear and distinctive explanation for each of the separate roles	In easy language - the listener: “is given information” - the co-thinker: “is asked to give opinion” - the advisor: “gives (un)solicited advice” - the partner: “works as an equal partner” - the decision-maker: “takes initiatives and/or makes decisions”

### Stage 3. The involvement matrix: roles of involvement in different phases of research

After having a provisional literature-based prototype for our tool, we wanted to make a formalized and feasible version, by incorporating the ideas, opinions and reflections of experience experts and those of health care professionals. Therefore, we organised a two-round focus group, discussing several topics: 1) the roles of involvement; 2) the language; and 3) the tool’s structure. The first round was preparatory and was arranged in group meetings. A total of 21 individuals were involved, with physical presence of patients (five young adults), relatives/parents of patients (*n* = 3), representatives of the patient organisation (*n* = 3), doctors (*n* = 2), and researchers (*n* = 3). The second round was confirmatory and was done by email with the same group, extended to include five more patients (three teenagers and two adults).

Regarding the roles, we paid attention to the number, variation, and names, and concluded that five distinguishable roles were satisfactory: listener, co-thinker, advisor, partner, and decision-maker. In relation to the language, we agreed that the tool should be easy in its explanations and its examples. For the structure of the tool, we decided that the tool could be a *matrix*, including not only the roles of involvement (horizontally) but also the phases of a research project (vertically). In this joint decision, we emphasized that the research phases should not be too fixed but rather general and flexible. Consequently, the tool could be used in most various projects.

Figure 1 shows

**Fig. 1:**
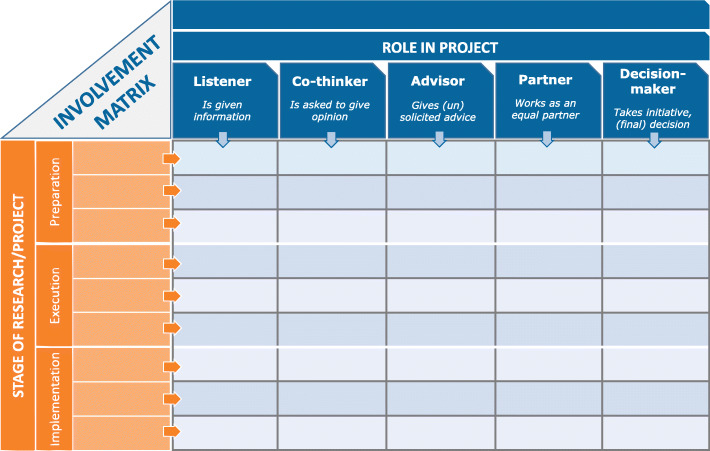
Involvement Matrix; www.kcrutrecht.nl/involvement-matrix. © Center of Excellence for Rehabilitation Medicine Utrecht, used with permission

the finalized “Involvement Matrix” [37] including a brief description of the roles of involvement and the phases of research.

### Formalisation of the involvement matrix

Based on the focus groups and confirmed by the first try-outs, we formalized that the Involvement Matrix is intended as a guide for the project leader or researcher to have dialogue with patients. Using the Matrix, agreements are made on the roles that the patient wishes to play and at which phase. In this way, the empty cells in the Matrix are filled up with concrete activities. Not all these details need to be finalised at once, this can be done as a step-by-step process (e.g. one activity per phase or sub-phase) throughout the project. We formalized the user’s guide, a user’s checklist, an overview with examples, and an animation, all co-created with experience experts. Thus, a total package for users was developed in addition to the Matrix itself, which is available at www.kcrutrecht.nl/involvement-matrix/ [38].

## Discussion

In collaboration with patient organizations, patients, parents, educators, doctors and researchers, we designed a tool to support patient and public involvement (PPI) in research projects. Our collaborative process resulted in the Involvement Matrix: a tool to support the conversation and discussion about roles and expectations on the individual level, aiming for authentic and sustainable partnerships in research. The Involvement Matrix includes five roles for involvement (Listener, Co-thinker, Advisor, Partner, and Decision-maker) over three main phases of research projects (Preparation, Execution, and Implementation).

Although patient and public involvement in research is expected, implementing PPI in practice can often prove challenging for all parties. Applying the Involvement Matrix before and during different phases of a project, has the potential to help researchers and patients to make clear agreements about research involvement and engagement of patients. The tool can be used prospectively, to discuss about possible roles of patients in different phases of projects, and retrospectively to discuss whether roles were carried out satisfactorily. First experiences with the Involvement Matrix are proving promising. The Matrix has already generated a lot of national and international interest by researchers and patient organizations [39].

The process of developing the Involvement Matrix was in itself a collaborative process, in which we learned from preceding steps. Not only for the tool development, but also in relation to wishes and expectations of researchers, patients and relatives on PPI in general. In this process, we discussed the value of participation in research, especially the importance of patients participating in the different roles, as described in the tool. There are several intrinsic values and reasons for PPI, namely from philosophical, political and quality perspectives [4, 5]. However, scientific evidence for PPI is still in its infancy. For instance, some argue the impact of PPI might need quantification. But at the same time, quantification is debatable here, given the discursive nature of PPI, and given the power relations between researchers and patients [40]. Furthermore, little evidence has yet been provided on the methods *how* to involve patients in research effectively. This methodological issue includes acknowledging the existence of various roles and expectations [2], which were the central topics in developing the present Involvement Matrix.

The main purpose of the Involvement Matrix is to facilitate the discussion between researchers and patients. Another important issue, however, is not addressed by this tool: the attitude towards PPI of researchers. Except for patient-driven research, the initiative for projects mostly lies with researchers. If they do not endorse the importance of PPI and if they are not motivated for PPI intrinsically, PPI will be limited. Funding agencies and policy makers could stimulate PPI by making the Involvement Matrix obligatory as a tool to qualify and quantify roles of patients in the research projects prospectively. However, no extrinsic motivation could supersede the intrinsic motivation needed for true collaboration.

The Involvement Matrix could and should be part of a larger set of implementation strategies for enhancing PPI and making PPI the norm in clinical research. Despite our intrinsic motivation of developing this tool collaboratively with patients and relatives, we experienced that PPI sometimes seems to stand in the way of our ‘regular/old’ ways of conducting research. Sometimes PPI takes more time and effort than just conducting research ‘on our own’. Using the Involvement Matrix as a continuous monitor will help forming a framework for PPI in all phases of a project.

### Next steps

As with all innovations and interventions, implementation is key to successful use. The Involvement Matrix is free to use and easy accessible [37, 38] and is being used in workshops, courses and individual training on PPI. Providing the Matrix in an instructional course for patients and researchers together could support PPI, and gives an opportunity to learn from each other and to learn from experiences in other projects.

The Involvement Matrix creates an opportunity for researchers and patients to prospectively discuss PPI in different stages of (research) projects using a structured approach. Further research should be directed at exploring other needs and wishes of both patients and researchers for bringing PPI in practice, including crucial elements as capabilities and motivation as agents for behavior change [41].

Finally, the field would benefit much from sharing and reporting on experiences [42]. Recently international evidence based, consensus informed guidelines have been published for reporting PPI in research (Guidance for Reporting Involvement of Patients and the Public; GRIPP2), including minimum reporting requirements in a short form [43, 44]. These guidelines aim to improve the quality, transparency, and consistency of the international PPI evidence base, to ensure PPI practice is based on the best evidence.

## Conclusion

In co-creation with patients and parents/relatives, health care professionals and researchers, the Involvement Matrix has been developed including a user’s guide. This tool aims to support PPI in research projects, focusing on role clarity and clarity on expectations. It can be used prospectively to discuss about possible roles of patients in different phases of projects, and retrospectively to discuss whether roles were carried out satisfactorily. Sharing experiences with the use of the Involvement Matrix and evaluating the impact of it are next steps in supporting patient involvement in research.

## Data Availability

Not applicable.

## Abbreviation

PPI: patient and public involvement
